# Intra- and inter-rater reliability of compressed breast thickness, applied force, and pressure distribution in screening mammography

**DOI:** 10.1177/20584601211062078

**Published:** 2021-12-09

**Authors:** Martina Voigt, Anetta Bolejko, Magnus Dustler

**Affiliations:** 1 Diagnostic Radiology, Department of Translational Medicine, Lund University, Skåne University Hospital, Malmö, Sweden; 2 Medical Radiation Physics, Department of Translational Medicine, Lund University, Skåne University Hospital, Malmö, Sweden

**Keywords:** Screening mammography, breast compression, reliability, radiography, radiographer

## Abstract

**Background:**

Ensuring equivalent and reproducible breast compression between mammographic screening rounds is important for the diagnostic performance of mammography, yet the extent to which screening mammography positioning and compression is reproducible for the individual woman is unknown.

**Purpose:**

To investigate the intra- and inter-rater reliability of breast compression in screening mammography.

**Materials and Methods:**

Eleven breast-healthy women participated in the study. Two experienced radiographers independently positioned and compressed the breasts of each participant in two projections—right craniocaudal and left mediolateral oblique—and at two time points. The spatial pressure distribution on the compressed breast was measured using a pressure sensor matrix. Applied force, compressed breast thickness, force in field of view, contact area, mean pressure, and center of mass (anterio-posterior and mediolateral axes) were measured. The reliabilities of the measures between the time points for each radiographer (intra-rater reliability) and between the radiographers (inter-rater reliability) were analyzed using the intraclass correlation coefficient (ICC).

**Results:**

Intra- and inter-rater reliabilities from both projections demonstrated good to excellent ICCs (≥0.82) for compressed breast thickness, contact area, and anterio-posterior center of mass. The other measures produced ICCs that varied from poor (≤0.42) to excellent (≥0.93) between time points and between radiographers.

**Conclusion:**

Intra- and inter-rater reliability of breast compression was consistently high for *compressed breast thickness*, *contact area*, and *anterio-posterior center of mass* but low for *mediolateral center of mass* and *applied force*. Further research is needed to establish objective and clinically useful parameters for the standardization of breast compression.

## Introduction

Breast cancer (BC) is the most common cancer among females worldwide,^1^ accounting for approximately 12% of female cancers in 2018, with around 2 000 000 women being diagnosed.^2^ Mammographic screening is recommended for BC detection at an asymptomatic stage of the disease, which enables early treatment and a reduction in BC mortality.^3,4^ Radiographers play an essential role in the mammographic screening program^5^ and are responsible for performing screening mammography examinations, which include positioning and compressing the woman’s breast.^5^ The diagnostic reading of mammograms is dependent on several factors, including image quality,^6^ which is majorly dependent on the radiographer’s positioning and compression of the breast during mammography acquisition.^7^ Furthermore, for the individual woman, an equivalent degree of breast compression over consecutive rounds of screening is important to facilitate correct diagnosis by allowing radiologists to more easily track parenchymal changes.^8^ Studies have shown that there are variations in how breast compression is applied by different radiographers,^9–16^ but the extent to which breast compression is reproducible for a given woman between screening rounds and how that may affect the interpretation of mammography is unknown.

Screening mammography consists of two bilateral projections of the breasts: right/left craniocaudal (CC) and right/left mediolateral oblique (MLO).^17^ The breast is compressed and held firmly with a compression paddle, which is intended to contribute to a clearer image of the breast tissue. Compression immobilizes the breast to minimize motion artifacts, increases the separation of parenchymal structures, and reduces the overall thickness. The thickness reduction reduces scattered radiation, thus lowering both radiation dose and image noise.^9^ If a breast is not sufficiently compressed, there is more tissue overlap and thus a higher risk of small abnormalities being obscured by overlying breast tissue.^18^

The radiographer’s application of breast compression is influenced by a subjective judgment that is based on experience and on breast characteristics like breast size and elasticity.^9^ Some women experience the breast compression as painful, which might also affect the amount of breast compression applied.^19^ The European guidelines for quality assurance in BC screening and diagnosis state that breast compression should be firmly applied but endurable, no higher than necessary to obtain good-quality images.^20^

An important goal in screening mammography is achieving equivalent breast compressions across consecutive screening rounds for each individual woman,^8^ but there is a lack of knowledge about the intra- and inter-rater reliability of breast compression and the factors affecting it. Breast compression has been investigated in terms of the use of compression force, compression pressure, and compressed breast thickness,^8^ and several studies have shown that the execution of breast compression varies among radiographers.^9–16^ A longitudinal mammogram assessment of three consecutive screening rounds at a breast center in the United Kingdom showed a significant variation in applied compression forces.^10^ Other studies conducted in the contexts of mammographic screening and diagnostic mammography (non-screening) in the United Kingdom and other countries have also found variations in compression forces applied by radiographers both within^11–14^ and between breast centers.^9,13–16^ It is also known that there are large variations in the distribution of pressure across the breast surface^21^ and that small changes in positioning can substantially affect this distribution.^22^ Similarly, substantial differences in pressure distribution have been observed when employing different compression paddle designs.^23^

Poor reliability of breast compression between screening rounds may result in variations in image quality, which may create a challenge for radiologists when tracking parenchymal changes over time. The aim of the current study was therefore to investigate the intra- and inter-rater reliability of breast positioning and compression in screening mammography.

## Materials and methods

This study was approved by the Swedish Ethical Review Authority (Dnr 2020–03652), with the purpose of exploring breast positioning variations as an addendum to a larger study investigating breast mechanical imaging (MI) as an adjunct to mammography screening. MI uses the distribution of material stress (reactive pressure) on the surface of the breast during mammographic compression to map the stiffness of the underlying tissue and derive additional diagnostic information.^24^ The larger MI study will include 1000 women recalled from screening.

### Sample

Participants were recruited via advertisement at the researchers’ workplaces: Skåne University Hospital (SUS), Malmö, Sweden. Female volunteers aged 18 or older were eligible. Exclusion criteria were women with breast implants, breast surgery in the last 6 months, ongoing work-up or treatment for BC or other breast disease, or poor command of Swedish. Signed informed consent was obtained before participation in the study.

### Measurements of breast compression and other data

Breast compression was performed using a single mammography unit, Senographe Pristina (GE Healthcare, Buc, France)^25^ using a FlexiForce compression paddle. A Tekscan CONFORMAT 5350 (Tekscan Inc., South Boston, MA, USA) body pressure measuring system (BPMS) pressure sensor was used. This sensor is designed for mapping the pressure distribution between the skin and materials such as cushions, mattresses, and seats; it has a 1 cm spatial resolution^26^ and has previously been used to investigate the distribution of breast compression pressure and to derive diagnostic information from such distributions—in other words, MI.^21–24^ In the present study, the sensor was used to investigate how pressure was distributed on the surface of the breast. Prior to data collection, the sensor was calibrated using a manufacturer-supplied vacuum calibration device.

Breast compression variables visible to the radiographer during breast compression were *applied force*, measured in decanewtons (daN), and *compressed breast thickness*, measured in millimeters (mm), for each projection. These were recorded from the display on the mammography unit. These two parameters are the only ones available for the radiographer in normal clinical practice.

**Figure 1.**
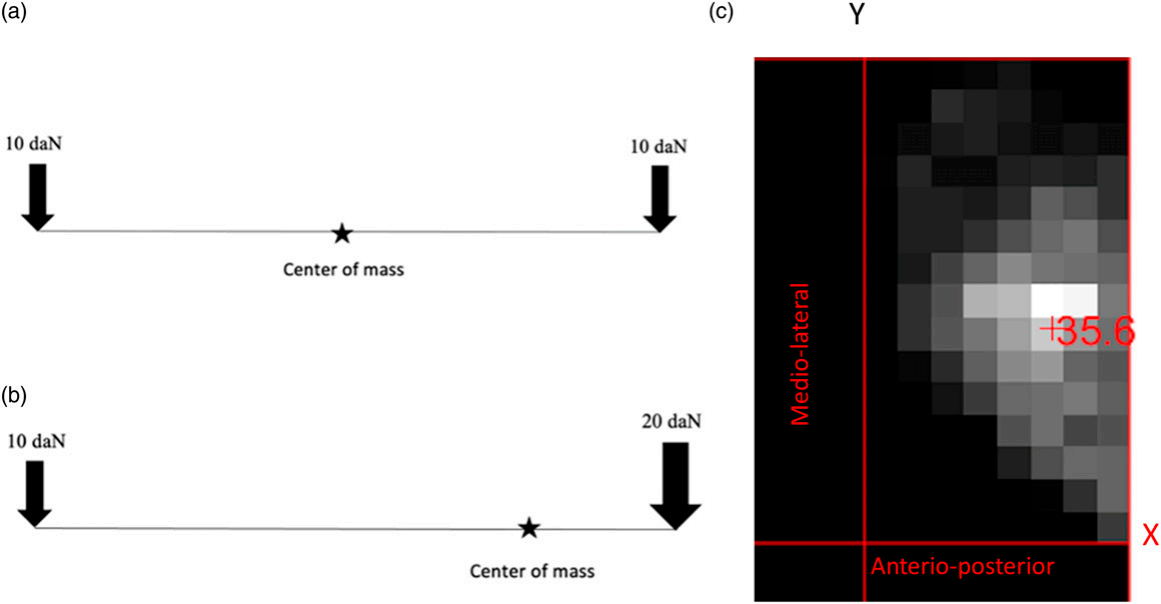
(a) 1D illustration of a lever with equally applied forces at each end and the center of mass (balance point) marked; (b) 1D illustration of a lever with unequally applied forces at each end—note that the center of mass shifts; and (c) image of a 2D pressure sensor output with breast in CC projection and an illustration of the coordinate system. The center of mass for the 2D sensor output is defined similarly along both axes.

The breast compression variables derived from the pressure distribution were the *force in field of view* (daN) (total force measured on the sensor) and the *contact area* between the breast and the sensor, measured in square centimeters (cm^2^). The *mean pressure* in kilopascals (kPa) was computed by dividing the *force in field of view* by the *contact area*. Additionally, the position of the *center of mass* of the pressure readings, in centimeters (cm), was determined. *Center of mass* is defined as the central point (balance point) of the recorded pressure distribution in the field of view and is defined along two axes, the anterio-posterior (x) axis and the mediolateral (y). The position of the center varies depending on how the force is distributed on the breast (Figure 1).

Additional characteristics of the sample were reported by the participants: age, height, weight (body mass index [BMI; kg/m^2^] was then calculated),^27^ ongoing or completed hormone replacement therapy, and brassiere cup size (as a substitute measure of breast size).

### Data collection

**Figure 2.**
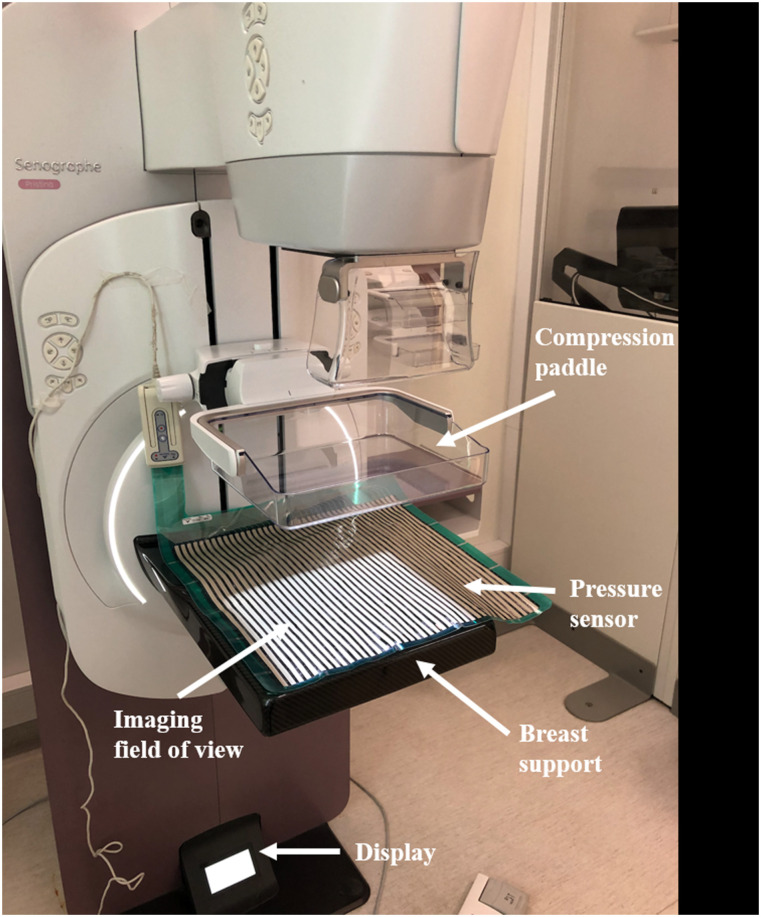
Image of a mammography unit and a pressure sensor placed on the breast support.

**Figure 3.**
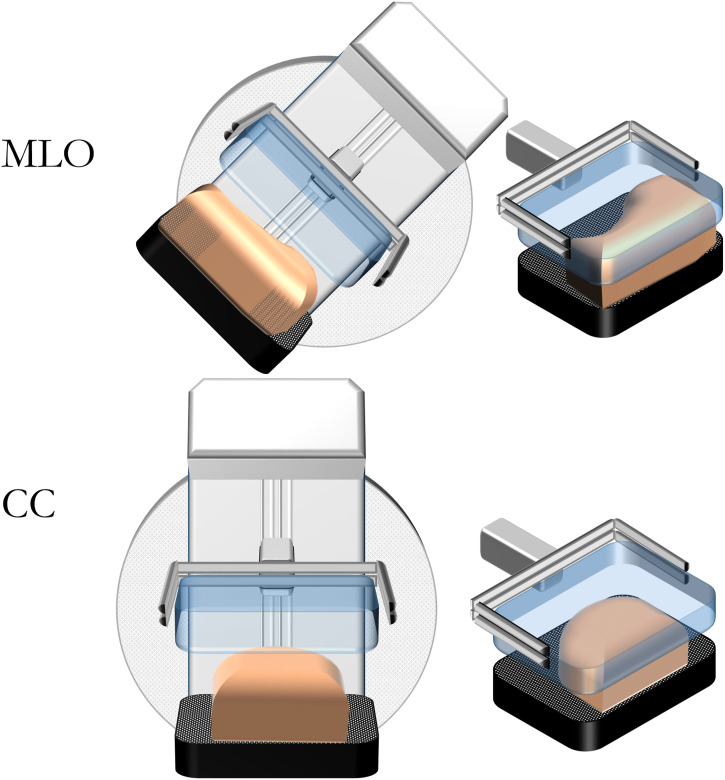
Illustration of a breast positioned in CC and MLO projections.

The data was collected at the Unilabs Breast Center at SUS Malmö. The pressure sensor was positioned on the breast support of the mammography unit (Figure 2) and placed in the image field of view, with one corner of the sensor in contact with one corner of the breast support (Figure 2). Two radiographers with more than 2 years of experience in screening mammography independently performed breast compressions of each participant in two stages. At the first stage (time point 1 [T1]), the first radiographer positioned and applied compression to the right breast of the participant in the CC projection (Figure 2) and then repeated with the left breast in the MLO projection (Figure 3); the same compressions were then performed in the same order by the second radiographer, who entered the room after the first radiographer was finished. At the second stage (time point 2 [T2]), which began approximately 10 min after completion of the first stage, both radiographers repeated the compressions in the same order for the same projections.

The participants were instructed to make no general comments on how the breast compression was experienced, unless they experienced pain. The purpose of this was to know if any compressions had to be conducted with less than normal compression force, but no pain was reported during the study. No mammographic images were acquired during the procedure. The pressure distribution recording was started just before the radiographer began the compression by lowering the compression paddle and ended immediately after the radiographer released the compression. One of the authors (MV), who is a registered radiographer and experienced in screening mammography, was present in the room for all data recording.

### Data analysis

The data was analyzed using IBM SPSS software version 27. Intraclass correlation coefficients (ICCs)^28,29^ were used to assess the intra- and inter-rater reliability of the study measures, and two-way, mixed-effects, single measures, absolute agreement ICCs were calculated for each. The following cut-off values for ICC reliabilities have been suggested: poor (less than 0.5), moderate (0.50–0.75), good (0.75–0.90), and excellent (>0.90).^28^

## Results

Eleven women participated in the study. The characteristics of the sample are presented in Table 1. The ICC analysis is presented in Tables 2 and 3. Descriptive numerical differences are presented in Table 4. Additional descriptive statistics of the measures are presented in the Supplementary File.

**Table 1.** Characteristics of the study sample.

Number of participants, n	11
Age in years, mean ± SD^a^	49.5 ± 11.7
BMI,^b^ n	
Normal weight, 18.5–24.9	6
Overweight, 25.0–29.9	3
Obese, ≥ 30	2
Hormone replacement therapy, n	
Yes	3
No	8
Bra cup size, n	
A	1
B	1
C	5
D	4

^a^= SD, standard deviation.

^b^= BMI, body mass index kg/m^2^ according to standard categories.^24^

**Table 2.** Intra-rater reliability of breast compression in CC projection and MLO projection; data presented for each radiographer between time points T1 and T2.

	Radiographer A CC/MLO		Radiographer B CC/MLO	
	ICC* (95% CI)	Correlation	ICC* (95% CI)	Correlation
Applied force (daN)	0.70 (0.23–0.91	Moderate	0.18 (−0.23–0.64)	Poor
	0.75 (0.31–0.93)	Good	0.67 (0.17–0.90)	Moderate
Compressed breast thickness (mm)	0.99 (0.96–1.00)	Excellent	0.98 (0.92–1.00)	Excellent
	0.96 (0.86–0.99)	Excellent	0.97 (0.85–0.99)	Excellent
Force in field of view (daN)	0.88 (0.61–0.97)	Good	0.74 (0.29–0.92)	Moderate
	0.41 (−0.22–0.80)	Poor	0.69 (0.20–0.91)	Moderate
Contact area (cm^2^)	0.95 (0.75–0.99)	Excellent	0.98 (0.94–1.00)	Excellent
	0.86 (0.56–0.96)	Good	0.98 (0.92–0.99)	Excellent
Mean pressure (kPa)	0.86 (0.57–0.96)	Good	0.64 (0.09–0.89)	Moderate
	0.56 (−0.01–0.86)	Moderate	0.35 (−0.30–0.77)	Poor
Anterio-posterior center of mass (cm)	0.96 (0.85–0.99)	Excellent	0.99 (0.93–1.00)	Excellent
	0.85 (0.46–0.96)	Good	0.99 (0.96–1.00)	Excellent
Mediolateral center of mass (cm)	−0.01 (−0.24–0.40)	Poor	0.52 (−0.04–0.84)	Moderate
	0.66 (0.16–0.90)	Moderate	0.82 (0.46–0.95)	Good

*Two-way mixed-effects, single measures, absolute agreement ICC model.

ICC: intraclass correlation coefficient; CI: confidence interval; CC: craniocaudal; MLO: mediolateral oblique; daN: decanewtons; mm: millimeters; cm: centimeters; kPa: kilopascals.

**Table 3.** Inter-rater reliability of breast compression between radiographers in CC projection and MLO projection; data presented for time points T1 and T2.

	Radiographer A versus Radiographer B			
	T1		T2	
	CC/MLO		CC/MLO	
	ICC* (95% CI)	Correlation	ICC* (95% CI)	Correlation
Applied force (daN)	0.52 (−0.13–0.85)	Moderate	−0.09 (−0.67–0.52)	Poor
	−0.51 (−0.87–0.14)	Poor	−0.82 (−1.07–0.24)	Poor
Compressed breast thickness (mm)	0.98 (0.92–1.00)	Excellent	0.97 (0.74–0.99)	Excellent
	0.97 (0.87–0.99)	Excellent	0.95 (0.91–0.99)	Excellent
Force in field of view (daN)	0.95 (0.81–0.99)	Excellent	0.79 (0.38–0.94)	Good
	0.42 (−0.11–0.79)	Poor	0.11 (−0.45–0.63)	Poor
Contact area (cm^2^)	0.94 (0.80–0.99)	Excellent	0.99 (0.98–1.00)	Excellent
	0.94 (0.48–0.99)	Excellent	0.82 (0.47–0.95)	Good
Mean pressure (kPa)	0.93 (0.76–0.98)	Excellent	0.54 (−0.05–0.85)	Moderate
	−0.18 (−0.71–0.46)	Poor	0.52 (−0.10–0.85)	Moderate
Anterio-posterior center of mass (cm)	0.95 (0.84–0.99)	Excellent	0.97 (0.89–0.99)	Excellent
	0.84 (0.15–0.96)	Good	0.92 (0.73–0.98)	Excellent
Mediolateral center of mass (cm)	0.05 (−0.44–0.58)	Poor	0.17 (−0.13–0.59)	Poor
	0.55 (−0.04–0.85)	Moderate	0.70 (0.22–0.91)	Moderate

*Two-way, mixed-effects, single measures, absolute agreement ICC model.

ICC: intraclass correlation coefficient; CI: confidence interval; CC: craniocaudal; MLO: mediolateral oblique; T1: time point 1; T2: time point 2; daN: decanewtons; mm: millimeters; cm: centimeters; kPa: kilopascals.

**Table 4.** Mean and standard deviation of intra- and inter-rater differences between measures; data pooled for readers (intra) and time points (inter).

	Applied force (daN)	Breast thickness (mm)	Force in field of view (N)	Contact area (cm^2^)	Mean pressure (kPa)	Anterio-posterior center of mass (cm)	Mediolateral center of mass (cm)
CC							
Intra	−0.5 ± 0.9	0.7 ± 2.1	−1.7 ± 9.5	−3.3 ± 8.8	−0.01 ± 1.6	0.02 ± 0.3	0.7 ± 1.9
Inter	−0.2 ± 1.2	1.8 ± 2.5	−0.04 ± 8.4	−2.2 ± 8.9	−0.1 ± 1.5	0.03 ± 0.3	−0.2 ± 2.0
MLO							
Intra	−0.05 ± 1.0	1.6 ± 3.9	−2.5 ± 10.7	−1.6 ± 13.5	−0.3 ± 1.3	0.2 ± 0.5	−0.7 ± 2.2
Inter	0.6 ± 2.2	−1.4 ± 4.1	−6.0 ± 12.9	−9.1 ± 14.3	−0.3 ± 1.5	0.3 ± 0.6	0.3 ± 0.6

CC: craniocaudal; MLO: mediolateral oblique; N: newtons; daN: decanewtons; mm: millimeters; cm: centimeters; kPa: kilopascals.

### Intra-rater reliability of breast compression

The intra-rater reliabilities of *compressed breast thickness*, *contact area*, and *anterio-posterior center of mass* were excellent (ICC ≥ 0.95) in the CC projection for both radiographers and good (near excellent) (ICC ≥ 0.85) in the MLO projection (Table 2). The remaining ICC values had mixed results, ranging from poor to good reliability (Table 2).

### Inter-rater reliability of breast compression

The inter-rater reliabilities of *compressed breast thickness*, *contact area*, and *anterio-posterior center of mass* were excellent (ICC ≥ 0.94) in the CC projection at both time points and good to excellent (ICC ≥ 0.82) in the MLO projection (Table 3). The remaining ICC values had mixed results, ranging from poor to excellent reliability (Table 3).

## Discussion

This study investigated the intra- and inter-rater reliability of breast compression in screening mammography among breast-healthy volunteers and found it to be consistently good to excellent (ICC ≥ 0.82) for *compressed breast thickness*, *contact area*, and *anterio-posterior center of mass*. The intra- and inter-rater reliabilities of the remainder ranged from poor (ICC ≤ 0.42) to excellent (ICC ≥ 0.93).

The results show that radiographers are not consistent in the application of compression force, which is consistent with previous studies in the contexts of mammographic screening and diagnostic mammography, which found variations in compression forces applied by radiographers within^10–14^ and between different breast clinics.^9,13–16^ The lack of objective and evidence-based guidelines regarding breast compression necessitates subjective judgments based on the interpretation and experience of the radiographer,^18^ which may explain the results. Nevertheless, although the intra- and inter-rater reliability of *applied force* was mainly poor to moderate, the reliability of *compressed breast thickness* was consistently excellent both among and between the radiographers. Similar results were found in a previous study, which showed no significant differences in *compressed breast thickness* by the same radiographer over three rounds of screening.^10^ Presumably, the excellent intra- and inter-rater reliability of *compressed breast thickness* may be explained by the radiographer’s consistent subjective assessment of the breast. Radiographers’ subjective breast compression behaviors^18^ and perceptions of mammography methods in mammographic screening and diagnostic mammography^30^ have also been investigated in qualitative studies, with some radiographers reporting that they applied breast compression based on breast characteristics rather than numerical values for *applied force*^18^—that is, a subjective assessment and perception of the breast tissue and its characteristics at compression influenced the breast compression.^18,30^ It is thus reasonable to argue that the radiographer’s subjective assessment of breast compression helped to provide the excellent intra- and inter-rater reliability of *compressed breast thickness* reported in our study.

Another finding of this study is the good to excellent intra-rater reliability of breast *contact area*, which supports the results from a previous study that used similar equipment to measure the distribution of surface pressure on the breast.^21^ The results also show good to excellent reliability of *anterio-posterior center of mass* and poor to moderate reliability of *mediolateral center of mass*, which seems to indicate that there is little variation in anterio-posterior positioning of the breast but that some displacement along the mediolateral axis can be expected. However, such variations do not seem to substantially affect the contact area between the breast and compression paddle, which supports the assumption that this has a minor effect on the distribution of pressure and thus on the quality of the breast compression.

From previous studies, it is known that compression pressure is often centered in the firmer juxtathoracic area of the posterior part of the breast, visible in the MLO projection, but less so on the central regions of the breast itself.^21–23^ It has been observed that partial exclusion of the juxtathoracic area affected pressure distribution and breast compression, displacing the center of mass anteriorly.^22^ Similarly, using a flexible compression paddle has also been shown to displace the center of mass anteriorly compared to the use of a standard, more rigid compression paddle.^23^ This suggests that, presumably, poor reliability of anterio-posterior positioning would have a greater detrimental effect than the observed mediolateral variability. Further studies in a larger sample and in the context of a mammographic screening program are needed to investigate the relationship between positioning and pressure distribution.

The results of this study have direct bearing on the repeatability and diagnostic accuracy of breast MI,^24^ which relies on measuring the spatial distribution of surface pressure on the compressed breast and relates it to the corresponding mammogram. Poor intra- and inter-rater reliability of breast compression that substantially affects the spatial distribution of pressure would detrimentally affect MI, but, as noted, variations occur mostly along the mediolateral axis. The resulting lateral shifts in the pressure distribution pattern would thus likely have only a minor effect on the diagnostic quality of MI; variations in the anterio-posterior axis would have a greater effect on MI, but are mostly absent; displaying mean variations that are much lower than the MI sensor’s spatial resolution of roughly 1 cm.

This study was not performed on women undergoing mammographic screening, which is a limitation, but the compression practices were identical to those used in screening mammography. In Sweden, BC screening is recommended for women aged 40–74 years, but in our study, adult women were included without age restriction. The age limit for BC screening can vary between countries, and it is known that breast density falls with age, being greatest among younger women, but the reliability of repeated breast compressions of the same woman is unlikely to be affected by density. The sample size of the study was small, but our results are in agreement with previous findings.^9–16,21–23^ A follow-up study including a larger sample of women of screening age would be valuable and could also allow sub-group analyses based on, for example, breast size and density.

The *mean pressure* in the study was computed approximately because force that is outside the sensor’s field of view is impossible to measure. It should be noted that the sensor has been investigated previously for its reproducibility and accuracy of measurement,^31^ and that there is no reason to believe that measurement error is the reason for the lower force on the breast compared to the applied force. Much of the compression force is absorbed in the juxtathoracic area.^21–23^

Image quality was not assessed in this study, which would have been a valuable complement to the investigation of intra- and inter-rater reliability of breast compression. However, it would have required radiation exposure, which is questionable for healthy volunteers. Further research investigating how various breast compression variables affect image quality and mammography reading is needed in order to develop objective guidelines and methods for breast compression in screening.

In conclusion, intra- and inter-rater reliability of breast compression was consistently high for *compressed breast thickness*, *contact area*, and *anterio-posterior center of mass* but low for *mediolateral center of mass* and *applied force*. For everyday practice, the results indicate that it is most important to focus on good positioning in the anterio-posterior axis. Further research is needed to establish objective and clinically useful parameters for the standardization of breast compression.

## Supplemental Material

sj-pdf-1-arr-10.1177_20584601211062078 – Supplemental Material for Intra- and inter-rater reliability of compressed breast thickness, applied force, and pressure distribution in screening mammography Click here for additional data file. Supplemental Material, sj-pdf-1-arr-10.1177_20584601211062078 for Intra- and inter-rater reliability of compressed breast thickness, applied force, and pressure distribution in screening mammography by Martina Voigt, Anetta Bolejko and Magnus Dustler in Acta Radiologica Open
