# Supplementary material for: Intra- and inter-rater reliability of compressed breast thickness, applied force, and pressure distribution in screening mammography
Source: Acta Radiol Open. 2021 Dec 9;10(12):20584601211062078. doi: 10.1177/20584601211062078 (PMC8819760; doi:10.1177/20584601211062078)
Supplement: sj-pdf-1-arr-10.1177_20584601211062078 – Supplemental Material for Intra- and inter-rater reliability of compressed breast thickness, applied force, and pressure distribution in screening mammography [file sj-pdf-1-arr-10.1177_20584601211062078.pdf]

Supplementary file

Tables 1 and 2 present descriptive statistics of the study measures.

Table 1. Breast compression values displayed on the mammography unit; data presented by each radiographer, projection and time points T1 and T2.

	Projection	Radiographer A<br>Mean ± SD<br>T1	 <br>T2	Radiographer B<br>Mean ± SD<br>T1	 <br>T2
Applied force (daN)	CC<br>MLO	10 ± 1<br>11 ± 1	10 ± 1<br>10 ± 1	10 ± 1<br>10 ± 1	11 ± 1<br>10 ± 1
Compressed breast thickness (mm)	CC<br>MLO	54 ± 14<br>56 ± 15	53 ± 13<br>55 ± 16	52 ± 13<br>58 ± 16	51 ± 12<br>56 ± 15

Values are presented as mean ± SD.
CC, craniocaudal; MLO, mediolateral oblique; SD, standard deviation; T1, time point 1; T2, time point 2; daN, decanewton; mm, millimetre.

Table 2. Breast compression values extracted from the pressure sensor; data presented by each radiographer, projection and time points T1 and T2.

	Projection	Radiographer A<br>Mean ± SD<br>T1	 <br>T2	Radiographer B<br>Mean ± SD<br>T1	 <br>T2
Force in field of view (daN)	CC<br>MLO	2.9 ± 1.5<br>2.7 ± 1.1	3.1 ± 1.8<br>3.0 ± 1.3	2.9 ± 1.4<br>3.3 ± 1.1	3.1 ± 1.6<br>3.5 ± 0.9
Contact area (cm^2)	CC<br>MLO	61.3 ± 31.5<br>84.8 ± 29.5	68.0 ± 37.5<br>85.4 ± 36.9	66.9 ± 38.7<br>92.9 ± 31.0	66.7 ± 39.5<br>95.5 ± 32.3
Mean pressure (kPa)	CC<br>MLO	5.2 ± 2.1<br>3.3 ± 1.2	4.8 ± 1.9<br>3.7 ± 1.9	5.0 ± 2.5<br>3.7 ± 0.8	5.3 ± 2.1<br>3.9 ± 1.0
Anterio-posterior center of mass (cm)	CC<br>MLO	36.6 ± 1.2<br>36.7 ± 1.1	36.6 ± 1.5<br>36.3 ± 1.4	36.5 ± 1.4<br>36.2 ± 1.4	36.6 ± 1.3<br>36.2 ± 1.5
Medio-lateral center of mass (cm)	CC<br>MLO	16.8 ± 1.7<br>12.6 ± 3.5	14.9 ± 0.8<br>14.1 ± 3.2	15.8 ± 1.2<br>13.2 ± 1.8	16.2 ± 1.0<br>13.0 ± 2.3

Values are presented as mean ± SD.
CC, craniocaudal; MLO, mediolateral oblique; SD, standard deviation; T1, time point 1; T2, time point 2; daN, decanewton; cm, centimetre; kPa, kilopascal.
